# Reliability of self‐report versus the capacity to consent to treatment instrument to make medical decisions in brain metastasis and other metastatic cancers

**DOI:** 10.1002/brb3.2303

**Published:** 2021-10-02

**Authors:** Mackenzie E. Fowler, Dario A. Marotta, Richard E. Kennedy, Adam Gerstenecker, Meredith Gammon, Kristen Triebel

**Affiliations:** ^1^ Department of Epidemiology School of Public Health University of Alabama at Birmingham Birmingham Alabama USA; ^2^ Division of Neuropsychology Department of Neurology University of Alabama at Birmingham Birmingham Alabama USA; ^3^ Alabama College of Osteopathic Medicine Dothan Alabama USA; ^4^ Division of Gerontology, Geriatrics, and Palliative Care Department of Medicine University of Alabama at Birmingham Birmingham Alabama USA; ^5^ O'Neal Comprehensive Cancer Center University of Alabama at Birmingham Birmingham Alabama USA

**Keywords:** brain metastasis, medical decision‐making capacity, metastatic cancer, predictors

## Abstract

**Objective:**

To evaluate the ability of persons with metastatic cancer to self‐assess their medical decision‐making capacity (MDC). To investigate this, we compared an objective measure of MDC with self‐ratings and evaluated predictors of agreement.

**Methods:**

Data were obtained from a cross‐sectional study of metastatic cancer patients at a large academic medical center. Across all standards of MDC, sensitivity, specificity, and reliability using Gwet's AC1 statistic were calculated using the objective measure as the gold standard. Logistic regression was used to evaluate predictors of agreement between the measures across all MDC standards.

**Results:**

In those with brain metastases, high sensitivity (greater than 0.7), but low specificity was observed for all standards. Poor reliability was observed across all standards. Higher age resulted in higher odds of disagreement for Standard 3 (*appreciation*) (OR: 1.07, 95% CI: 1.00, 1.15) and Standard 4 (*reasoning*) (OR: 1.05, 95% CI: 1.00, 1.10). For Standard 3, chemotherapy use and brain metastases compared to other metastases resulted in higher odds of disagreement (Chemotherapy: OR: 5.62, 95% CI: 1.37, 23.09, Brain Metastases: OR: 5.93, 95% CI: 1.28, 27.55). For Standard 5 (*understanding)*, no predictors were associated with disagreement.

**Conclusions:**

For less cognitively complex standards (e.g., appreciation), self‐report may be more valid and reliable than more cognitively complex standards (e.g., reasoning or understanding). However, overall, MDC self‐report in the current sample is suboptimal. Thus, the need for detailed assessment of MDC, especially when patients are older or used chemotherapy, is indicated. Other studies should be conducted to assess MDC agreement longitudinally.

## 1 INTRODUCTION

Medical decision‐making capacity (MDC) is a cognitively mediated functional ability referring to the ability to make informed decisions about medical treatment (Gerstenecker, Niccolai, Marson, & Triebel, 2016). Four core standards of MDC have been outlined in the research literature (Marson, Ingram, Cody, & Harrell, 1995) and include expressing choice, appreciation, reasoning, and understanding. MDC represents an important function at all life stages, but particularly in patients with serious illness, such as metastatic cancer. Take, for instance, the standard of *understanding* and a person with brain cancer, treatment choices include whole‐brain radiation therapy and stereotactic radiation. *Understanding* allows for recall and conceptual knowledge about each treatment option and its associated advantages and disadvantages. Without this knowledge, informed medical decisions cannot be made. MDC has also been described as a sliding scale in which risks of the decision play a role in determining the level of competency (Drane, 1984). For a lower risk and less complex decision, the standard for competency is lower. However, for a higher risk and more complex decision, the standard for competency is higher. Decisions about cancer treatment will likely fall into the latter category, but metastatic cancer patients will also face many other less risky decisions throughout their care. Therefore, it is important to understand the levels of MDC according to various standards in patients with metastatic cancer.

In adults with primary cancer, approximately 9% will experience symptomatic brain metastases (Eichler et al., 2011), representing the most common type of central nervous system tumors in the United States (Ostrom, Wright, & Barnholtz‐Sloan, 2018). Brain metastases, like other forms of cancer, are associated with a range of symptoms (Marotta et al., 2020), many of which occur before beginning treatment (Janelsins, Kesler, Ahles, & Morrow, 2014). Many studies indicate that metastatic cancer in general accounts for 90% of cancer deaths (Chambers, Groom, & MacDonald, 2002; Jean‐Pierre & McDonald, 2016). Aggressive treatment strategies are available and often consist of a combination of surgery, radiation, and pharmacologic interventions (Kotecha, Gondi, Ahluwalia, Brastianos, & Mehta, 2018). However, although medical management increases life expectancy, it is associated with a number of side effects, including cognitive impairment (Janelsins et al., 2014; Jean‐Pierre & McDonald, 2016; Lange et al., 2019). In multiple studies, our group demonstrated that this cognitive impairment leads to corresponding impairment in MDC (Gerstenecker et al., 2020; Triebel et al., 2015).

While MDC has been evaluated in persons with metastatic cancer using subjective, objective, and informant measures, the agreement between these measures has not been evaluated. For instance, persons with cognitive decline in metastatic cancer often do not perform poorly on objective measures (Lange et al., 2019). In turn, some persons with metastatic cancer may be mistakenly identified as possessing fully intact MDC when, in fact, they pose risk for making poor and uninformed treatment decisions. A self‐reported or informant‐reported measure may indicate intact MDC based on everyday behavior and general functioning, but performance when faced with an actual decision may be lacking due to subtle cognitive changes not necessarily observable to the patient or informant.

To examine the gap between self‐rated MDC and objective MDC, we administered a self‐report measure of MDC to a group of persons with metastatic cancer that has spread to the brain and to a group with metastatic cancer that has not spread to the brain. We then compared these self‐report ratings of MDC to a performance‐based measure of MDC. We also investigated demographic and clinical correlates of agreement between self‐reported and objectively measured MDC in both groups. We hypothesized that self‐report would be less reliable in determining MDC compared to the objective measure.

## 2 METHODS

### 2.1 Participants

Newly diagnosed (i.e., first diagnosis and first treatment) persons with metastatic cancer were recruited from the Departments of Radiation Oncology, Medical Oncology, or Neurosurgery at the University of Alabama at Birmingham (UAB) between the dates of August 2011 and December 2018. Two groups were recruited: those with metastasis to the brain and those with metastasis not to the brain. All diagnoses were made by a board‐certified oncologist and primary tumors were verified histologically. Inclusion criteria were as follows: age ≥ 19 years; presence of a supratentorial lesion (for the brain metastasis group); and absence of pre‐existing psychiatric (mild depression or anxiety symptoms not exclusionary), neurologic, or medical illness.

Participants with a Six‐Item Cognitive Screening (Callahan, Unverzagt, Hui, Perkins, & Hendrie, 2002) score of ≥ 3 were included. Participants were assessed prior to or within a week of starting radiation therapy. This study was approved by the institutional review board at UAB (Approval Number: X141023002). Written informed consent was obtained from either the patient or legally authorized representative prior to data collection. The data supporting the study findings are available on request from the corresponding author. The data are not publicly available due to privacy or ethical restrictions.

The following treatments for brain metastases were used: conventional surgery; single fraction radiosurgery with Gamma Knife or Linear Accelerator (LINAC) technology (15 Gy–24 Gy) for tumors ≤4 cm; hypofractionated focal radiation with LINAC for tumors >3–4 cm (5–6 Gy x 5 fractions for 25–30 Gy total); and whole brain radiation therapy (WBRT) (with LINAC technology) (30 Gy in 10 fractions to 37.5 Gy in 15 fractions). Off‐study guidelines for radiosurgical treatment at UAB followed maximum tolerated doses outlined in the Radiation Therapy Oncology Group (RTOG) 9005 (Shaw et al., 2000). A majority of patients had previously received chemotherapy. Twenty‐nine patients were actively receiving chemotherapy at the time of this study.

### 2.2 Procedures

Data were collected during a single visit. A comprehensive neuropsychological battery was administered, which included assessments of: (1) executive functioning (Trail Making Test Part B) (Reitan & Wolfson, 1993); (2) verbal learning and memory (Hopkins Verbal Learning Test Revised [HVLT]) (Brandt & Benedict, 2001); (3) attention (Wechsler Adult Intelligence Scale‐Third Edition (WAIS‐III) Digit Span subtest) (Wechsler, 1997); (4) processing speed (Trail Making Test Part A and WAIS‐III Digital Symbol Coding subtest) (Reitan & Wolfson, 1993; Wechsler, 1997); and (5) verbal fluency (Controlled Oral Word Association test) (Benton & Hamsher, 1989). Self‐report measures of mood (Hospital Anxiety and Depression Scale) (Bjelland, Dahl, Haug, & Neckelmann, 2002) and quality of life (Functional Assessment of Cancer Therapy‐General Version 4) (Cella et al., 1993) were also administered. Overall, the entire battery required about 2 hours to complete. No participants refused to complete the test battery. Trained psychometrists administered the study measures and quality control was conducted by a board‐certified neuropsychologist (KT). Medical and treatment data were extracted from medical records.

### 2.3 Capacity to Consent to Treatment Instrument

The Capacity to Consent to Treatment Instrument (CCTI) is a reliable and valid performance‐based measure designed to evaluate MDC (Gerstenecker et al., 2016; Marson et al., 1995; Triebel et al., 2015) using five legal standards: Standard 1: expressing a treatment choice (*expressing choice*), Standard 2: making the reasonable choice based on provided information (*making choice*), Standard 3: appreciating the personal consequences of the choice (*appreciation*), Standard 4: providing rational reasons for treatment choice (*reasoning*), and Standard 5: understanding the situation, choices, and risks/benefits of each (*understanding*). The Standards are a hierarchical progression where increasing Standards represent more complex abilities and fully encompass the definition of MDC. The instrument contains a vignette of a hypothetical medical situation, including symptoms and two treatment options along with their risks and benefits. The vignette is presented orally and in writing. Following initial presentation, the written form is withdrawn and a series of standardized oral questions are asked to assess the four core standards of consent (*expressing choice, appreciation, reasoning, and understanding*) (Grisso, 1986; Marson et al., 1995). The CCTI has been used in multiple prior studies across a range of diagnoses, including mild cognitive impairment and Alzheimer's disease (Marson et al., 1995; Okonkwo et al., 2007), Parkinson's disease (Martin et al., 2008), and cancer (including metastasis) (Gerstenecker et al., 2020; Martin, Gerstenecker, Nabors, Marson, & Triebel, 2015; Triebel et al., 2015).

### 2.4 Current Medical Decision‐Making Capacity Rating Self‐Report Version

The Current Medical Decision‐Making Capacity Rating (CMDC) Self‐Report Version is a recently developed self‐report measure of MDC. Participants are presented with an example treatment scenario and then queried using seven questions to determine their perception of their own overall MDC and their MDC within each specific standard. For example, Question 3 asks, “*Are you able to appreciate the personal consequences of making a medical treatment choice? That is, do you understand the possible consequences to you personally of one choice versus the other choice?”* This question aims to evaluate a participant's assessment of their own ability in Standard 3 (*appreciation)*. Answer choices for this and similar questions evaluating overall MDC and specifically Standards 4 and 5 (*reasoning* and *understanding*, respectively) include “Yes‐without any help,” “Yes‐but I need help with this,” and “No‐I am unable to do this.” For this analysis, the latter two responses were considered as “impaired” on self‐reported MDC. The measure is included in the Supplemenary material.

### 2.5 Demographics

Self‐reported age, gender, race, years of education, and marital status were collected.

### 2.6 Cancer care

Information regarding participants’ cancer care was extracted from the medical record by research assistants using standardized data collection forms. Such data included type of primary cancer, radiation and type of radiation, surgical resection, chemotherapy, and hormone treatment.

### 2.7 Medications

Current prescription medications were collected by self‐report.

### 2.8 Comorbidities

Potentially relevant comorbidities were also collected via self‐report, including diabetes, learning disorders, or head injuries.

### 2.9 Statistical analyses

Bivariate analyses using chi‐square tests and *t*‐tests for categorical and continuous variables, respectively, were performed to evaluate differences between those impaired versus those intact on the CCTI within the overall study group and separately for the brain metastasis and other metastasis group. Validity of the self‐report of MDC was measured via sensitivity, specificity, positive predictive value, and negative predictive value using the CCTI measure as the gold‐standard. Validity was assessed overall and in each type of metastasis. In this analysis, sensitivity indicated the probability of self‐reporting intact given being intact on the CCTI. Specificity indicated the probability of self‐reporting impaired given being impaired on the CCTI. Positive predictive value indicated the probability of being intact on the CCTI given self‐report of intact, and negative predictive value indicated the probability of being impaired on the CCTI given self‐report of impaired. Additionally, reliability of the self‐report measure compared to the CCTI was performed using Gwet's AC1 statistic (Gwet, 2008) in both metastasis groups and overall. Finally, two‐stage logistic regression modeling was used to evaluate possible predictors of agreement with a jackknife estimate of variance (Lipsitz, Parzen, Fitzmaurice, & Klar, 2003). Predictors included age, race, sex, education, past chemotherapy use, TRAILS B t‐score, HVLT total recall t‐score, total number of impaired neuropsychological tests, and cancer type. All analyses were assessed at the α = 0.05 significance level and conducted in SAS Version 9.4 (SAS Institute, Inc., Cary, NC) and RStudio Version 1.1.423 (R Foundation) using the *epiR* package (Stevenson et al., 2018). Gwet's AC1 statistic was evaluated using the *AC1* function in R (https://cran.r‐project.org/web/packages/rel/rel.pdf).

## 3 RESULTS

The overall sample consisted of 155 participants with 114 having brain metastasis and 41 having metastasis to other sites. Overall and by metastasis group, the majority of the sample self‐reported as intact (Overall: 78.7%, Brain Metastasis: 74.4%, and Other Metastasis: 89.2%). No differences were observed in demographics, comorbidities, or clinical variables between those with intact versus impaired CCTI scores (Table 1). However, differences in neuropsychological testing were abundant. Those who were intact on CCTI Standards 3 (*appreciation)*, 4 (*reasoning)*, and 5 (*understanding)* had higher scores on measures of attention/working memory, verbal fluency, verbal memory, and executive functioning (Table S1). Those intact on Standards 3–5 also had lower total number of neuropsychological tests in the impaired range (Table 1). This pattern followed when conducting bivariate analyses among those with brain metastasis only, but when examining bivariate statistics among those with other metastases, no significant differences were seen for any variables with exception of category fluencies.

**TABLE 1:** Participant characteristics by objective medical decision‐making capacity performance and diagnosis^a^

	Overall (*n* = 155)			Brain metastasis (*n* = 114)			Other metastasis (*n* = 41)		
Variable	*Intact*	*Impaired*	*p*‐value	*Intact*	*Impaired*	*p*‐value	*Intact*	*Impaired*	*p*‐value
Demographics									
*Age*	57.6 ± 11.8	59.9 ± 12.1	.2430	56.8 ± 12.3	60.0 ± 11.6	.1651	59.4 ± 10.9	59.6 ± 13.6	.9550
*Education*	14.0 ± 2.5	13.2 ± 2.5	.0563	14.1 ± 2.6	13.0 ± 2.7	.0533	13.8 ± 2.2	13.6 ± 1.9	.7838
*Sex, male*	29 (46.8)	45 (48.4)	.8439	18 (42.9)	35 (48.6)	.5524	8 (40.0)	9 (42.9)	.8527
*Race, White*	49 (79.0)	71 (76.3)	.6696	34 (81.0)	56 (77.8)	.6320	15 (75.0)	15 (71.4)	.7964
Comorbidities									
*Seizure*	7 (11.5)	13 (14.6)	.5795	7 (17.1)	13 (19.1)	.7894	0 (0)	1 (4.8)	1.0000
*Psychiatric illness*	1 (2.4)	4 (6.2)	.6462	0 (0)	4 (9.1)	.2925	1 (5.0)	0 (0)	.4878
*Learning disorders*	1 (2.4)	3 (4.6)	1.0000	1 (4.6)	2 (4.6)	1.0000	0 (0)	1 (4.8)	1.0000
*Head injury*	2 (4.8)	5 (7.7)	.7019	1 (4.6)	4 (9.1)	.6577	1 (5.0)	1 (4.8)	1.0000
Clinical variables									
*Type of radiation*			.4866			.4783			.7125
Focal Gamma Knife	12 (20.3)	20 (21.7)		12 (28.6)	19 (26.4)		0 (0)	1 (5.0)	
Focal SRS	35 (59.3)	47 (51.1)		23 (54.8)	31 (43.1)		12 (70.6)	16 (80.0)	
Focal other	2 (3.4)	1 (1.1)		0 (0)	0 (0)		2 (11.8)	1 (5.0)	
WBRT	7 (11.9)	19 (20.7)		7 (16.7)	19 (26.4)		0 (0)	0 (0)	
Both focal and WBRT	3 (5.1)	3 (3.3)		0 (0)	2 (2.8)		0 (0)	0 (0)	
None	0 (0)	2 (2.2)		0 (0)	1 (1.4)		3 (17.7)	2 (10.0)	
*Surgical resection*	9 (14.8)	13 (14.0)	.8930	9 (21.4)	11 (15.3)	.4049	0	2 (9.5)	.4885
*Prior chemotherapy*	41 (66.1)	50 (54.4)	.1448	26 (61.9)	33 (46.5)	.1126	15 (75.0)	17 (81.0)	.7186
*Present chemotherapy*	15 (24.2)	14 (15.1)	.1529	7 (16.7)	4 (5.6)	.0957	8 (40.0)	10 (47.6)	.6232
*Present hormone treatment*	7 (14.3)	8 (11.1)	.7796	5 (17.2)	3 (5.9)	.1311	2 (10.0)	5 (23.8)	.4099
*Subjective memory/cognitive changes*	13 (31.0)	32 (49.2)	.0731	5 (22.7)	18 (40.9)	.1778	8 (40.0)	14 (66.7)	.0870
*HVLT Total Recall t‐score*	43.7 (11.4)	36.2 (11.8)	**.0002**	45.3 (12.8)	38.4 (12.1)	**.0097**	47.0 (10.9)	45.3 (11.0)	.6311
*Trails B t‐score*	45.9 (12.1)	40.1 (12.1)	**.0068**	41.8 (11.5)	34.6 (11.5)	**.0020**	47.6 (10.4)	41.6 (11.6)	.0906
*Total neuropsychological tests impaired*	1.3 ± 1.7	2.7 ± 2.4	**.0002**	1.6 ± 1.8	3.1 ± 2.5	**.0026**	0.9 ± 1.5	1.5 ± 1.5	.1893

^a^ Intact versus impaired status determined via performance on Standards 3–5 on the Capacity to Consent to Treatment Index (CCTI). Bold values indicate statistical significance.

### 3.1 Overall study group agreement

Analyses of validity and reliability revealed differences across CCTI standards. Evaluation for Standards 3–5 overall indicated high sensitivity and high negative predictive value (greater than 0.7) for the overall study sample, but low specificity and positive predictive value (Table 2). These results indicate that those identifying as intact on the CCTI were likely to self‐report being intact, but those impaired on the CCTI are unlikely to self‐report as impaired. Overall for each standard, the self‐rated measure correctly identifies participants given they are intact on the CCTI. Sensitivity and specificity results were similar for Standards 3 (*appreciation)*, 4 (*reasoning)*, and 5 (*understanding)*, but for Standard 3, positive predictive value was high and negative predictive value was low (Table 2). When examining reliability, however, the Gwet's AC1 statistic for all groups and standards remained low (Table 2). This indicates that the ability of the self‐rated measure to correctly identify participants as impaired or intact is inconsistent compared to the objective measure.

**TABLE 2:** Reliability and validity of self‐report medical decision‐making incapacity compared to Capacity to Consent to Treatment Instrument (CCTI)^a^

Variable	Gwet's AC1 Agreement Statistic (95% CI)	Sensitivity (95% CI)	Specificity (95% CI)	Positive predictive value (PPV) [95% CI]	Negative predictive value (NPV) [95% CI]
Standards 3–5					
Overall	0.16 (0, 0.34)	0.91 (0.80, 0.97)	0.30 (0.20, 0.42)	0.49 (0.39, 0.59)	0.81 (0.62, 0.94)
Brain metastasis	0.15 (0, 0.35)	0.91 (0.76, 0.98)	0.36 (0.23, 0.50)	0.46 (0.34, 0.59)	0.87 (0.66, 0.97)
Other metastasis	0.23 (0, 0.58)	0.90 (0.68, 0.99)	0.12 (0.01, 0.36)	0.55 (0.36, 0.72)	0.50 (0.07, 0.93)
Standard 3					
Overall	0.70 (0.58, 0.81)	0.80 (0.71, 0.87)	0.25 (0.03, 0.65)	0.93 (0.86, 0.98)	0.09 (0.01, 0.28)
Brain metastasis	0.64 (0.47, 0.81)	0.76 (0.64, 0.85)	0.40 (0.05, 0.85)	0.95 (0.85, 0.99)	0.11 (0.01, 0.33)
Other metastasis	0.77 (0.59, 0.95)	0.88 (0.73, 0.97)	0 (0–0.71)	0.91 (0.76, 0.98)	0 (0, 0.60)
Standard 4					
Overall	0.40 (0.22, 0.58)	0.83 (0.73, 0.91)	0.24 (0.11, 0.40)	0.68 (0.58, 0.78)	0.41 (0.21, 0.64)
Brain metastasis	0.36 (0.13, 0.58)	0.78 (0.65, 0.89)	0.31 (0.14, 0.52)	0.69 (0.55, 0.80)	0.42 (0.20, 0.67)
Other metastasis	0.48 (0.19, 0.78)	0.92 (0.74, 0.99)	0.08 (0, 0.38)	0.68 (0.49, 0.83)	0.33 (0.01, 0.91)
Standard 5					
Overall	0.26 (0.07, 0.45)	0.74 (0.62, 0.83)	0.33 (0.20, 0.50)	0.65 (0.54, 0.76)	0.42 (0.25, 0.61)
Brain metastasis	0.15 (0, 0.38)	0.67 (0.51, 0.81)	0.41 (0.25, 0.59)	0.59 (0.44, 0.73)	0.50 (0.31, 0.69)
Other metastasis	0.51 (0.22, 0.79)	0.83 (0.64, 0.94)	0 (0, 0.37)	0.75 (0.57, 0.89)	0 (0, 0.52)

^a^ Assessed at α = 0.05 significant level; Standard 3 = appreciation, Standard 4 = reasoning; Standard 5 = understanding.

### 3.2 Brain metastasis group agreement

Standards 3–5 overall in the brain metastasis group indicated high sensitivity and negative predictive value (greater than 0.7), but low specificity and positive predictive value (less than 0.7) (Table 2). When examining each CCTI standard, Standard 3 (*appreciation)* indicated high sensitivity and positive predictive value but low specificity and negative predictive value. Standard 4 (*reasoning)* revealed a similar pattern, but the positive predictive value was slightly lower at 0.69 (95% CI: 0.55, 0.80). Standard 5 (*understanding)* also revealed a similar pattern, but with both positive predictive value (0.59, 95% CI: 0.44, 0.73) and negative predictive value (0.50, 95% CI: 0.31, 0.69) being slightly lower. These results again indicate that persons with brain metastasis who test as intact on the CCTI are likely to self‐report being intact, but that persons with brain metastasis who test as impaired on the CCTI are unlikely to self‐report as impaired. However, concordance between testing results and self‐report was lower for the more complex standards of *reasoning* and *understanding*. Gwet's AC1 statistic of reliability was low for all Standards, combined and separately, and indicated low reliability/consistency for the self‐report measure compared to CCTI (Table 2).

### 3.3 Other metastasis group agreement

The other metastasis group revealed high sensitivity, but low specificity, positive predictive value, and negative predictive value for Standards 3–5 overall (Table 2). Separating each standard resulted in slightly different results. For Standard 3 (*appreciation)*, high sensitivity and positive predictive value was observed, but zero specificity and negative predictive value was observed. Standard 4 (*reasoning)* revealed similar results with slightly lower positive predictive value (0.68, 95% CI: 0.49, 0.83) and slightly higher negative predictive value (0.33, 95% CI: 0.01, 0.91). Standard 5 (*understanding)* revealed results akin to Standard 3. Overall, however, these results indicate that in the other metastasis group those who tested as intact on the CCTI were likely to self‐report as intact, but unlikely to self‐report as impaired if testing impaired. The Gwet's AC1 statistic remained low for all Standards with exception of Standard 3 (0.77, 95% CI: 0.59, 0.95) (Table 2), thus indicating lack of consistency between self‐report of MDC and actual MDC performance. The less complex standard of *appreciation* appeared to have better consistency for self‐report than the more complex standards. For all Standards overall and individually, the other metastasis group exhibited higher reliability and sensitivity, but lower specificity than the brain metastasis group.

### 3.4 Predictors of agreement

When examining predictors of agreement for the overall study group, a 1‐year increase in age resulted in 7% increased odds of disagreement for Standard 3 alone (95% CI: 1.00, 1.15) and a 5% increased odds of disagreement for Standard 4 alone (95% CI: 1.00, 1.10). For Standard 3 (*appreciation)* alone, chemotherapy use resulted in a 5.62‐fold increased odds of disagreement, but this estimate had large variability (95% CI: 1.37, 23.09). Also for Standard 3, brain metastasis resulted in a 5.93‐fold increased odds of disagreement compared to other metastases, but again this estimate had wide variability (95% CI: 1.28, 27.55). For Standards 3–5 overall and in Standard 5 (*understanding)* alone, no predictors were associated with disagreement (Table 3).

**TABLE 3:** Odds ratios (OR) and 95% confidence intervals (95% CI) for disagreement in each standard of the Capacity to Consent to Treatment Instrument in brain metastasis patients^a^

Predictor	Standards 3–5	Standard 3	Standard 4	Standard 5
Age, years	**1.04 (1.00, 1.09)**	**1.07 (1.00, 1.15)**	**1.05 (1.00, 1.10)**	0.99 (0.95, 1.04)
Education, years	0.96 (0.77, 1.20)	0.81 (0.58, 1.12)	0.96 (0.75, 1.23)	0.84 (0.66, 1.06)
Sex, female	1.72 (0.60, 4.95)	0.79 (0.17, 3.64)	2.28 (0.66, 7.88)	2.08 (0.68, 6.36)
Race, White	1.70 (0.46, 6.27)	0.28 (0.05, 1.58)	1.77 (0.38, 8.18)	0.85 (0.20, 3.68)
Past chemotherapy	0.28 (0.09, 0.84)	**5.62 (1.37, 23.09)**	0.33 (0.09, 1.20)	0.65 (0.19, 2.15)
TRAILS B t‐score	1.03 (0.97, 1.08)	1.01 (0.93, 1.08)	0.94 (0.88, 1.00)	1.03 (0.97, 1.09)
HVLT Total Recall t‐score	0.95 (0.89, 1.01)	0.99 (0.91, 1.07)	0.97 (0.90, 1.04)	1.02 (0.95, 1.10)
Total impaired neuropsychological tests	1.14 (0.72, 1.81)	1.07 (0.64, 1.81)	1.07 (0.66, 1.72)	1.33 (0.82, 2.15)
Cancer type, brain metastasis	0.57 (0.18, 1.81)	**5.93 (1.28, 27.55)**	0.67 (0.17, 2.58)	3.49 (0.99, 12.37)

^a^ Assessed at the α = 0.05 significance level using two‐stage logistic regression with a jackknife estimator of variance. Bold values indicate statistical significance.

Abbreviation: HVLT, Hopkins Verbal Learning Test.

## 4 DISCUSSION

MDC has been studied across several diseases, but, to our knowledge, no studies have examined the ability of persons with brain metastases to report and recognize their own MDC. To address this gap, the current study evaluated self‐report of MDC among persons with metastatic cancer. Investigations into the insight of MDC in persons with metastatic cancer are important because they can help clinicians in determining how much weight to give self‐reports of MDC. Overall, our results showed that self‐reports were primarily valid only when a participant demonstrated intact MDC on an objective measure. For those with impaired MDC, concordance between self‐report and actual MDC performance was low. Taken together, these results indicate suboptimal reliability of our self‐report measure. Said another way, as a whole, self‐report of MDC in our sample of persons with metastatic cancer was a poor indicator of actual MDC performance.

Although overall reliability of our self‐report measure was low, reliability varied according to the cognitive complexity of each MDC standard. For the less cognitively complex Standard of *appreciation*, reliability of self‐report was high. However, for the more cognitively complex Standards of *reasoning* and *understanding*, participants were less likely to correctly self‐report as intact. Thus, patients may be basing judgment of their own MDC on less complex tasks, such as the ability to remember presented information, while possessing less insight into difficulty with more complex MDC tasks, such as being able to logically understand presented information. Thus, these results imply that simply asking a participant with cancer metastasis, regardless of site, whether they feel confident in their ability to make their own medical decisions may not be sufficient and more formal measures, such as the CCTI, may be warranted. While this requires a psychological or psychiatric referral, it is important to ascertain whether participants fully understand treatment options and their implications both from a legal and an ethical standpoint.

Although both study groups demonstrated difficulty correctly identifying level of performance on the CCTI, an interesting pattern was observed across our study groups. For the other metastasis group, the ability to correctly identify as intact was better than for the brain metastasis group. However, the brain metastasis group was slightly better than the other metastasis group at correctly identifying as impaired. These findings in the other metastasis group are not surprising considering the cognitive impact associated with brain metastasis (Gerstenecker et al., 2014). However, the finding in the brain metastasis group was unexpected. This likely indicates that persons with brain metastasis possess some awareness that their condition is detrimental to cognitive ability and ultimately the ability to make sound medical decisions. Conversely, the more subtle cognitive deficits seen in persons with metastasis that has not spread to the brain (Lange et al., 2019) may lead to a false sense of security when it comes to estimating MDC in the patient group.

Overall, self‐report was more reliable in the other metastasis group than in the brain metastasis group. However, specificity remained low even in the other metastasis group, indicating that self‐report of MDC may not be valid in metastatic cancer, regardless of spread to the brain. Moreover, when analyzing the study sample as a whole, most study participants self‐identified as intact, and this may have driven some of the discordance seen in the results where many who were impaired on the CCTI self‐rated as intact simply due to the high proportion of intact self‐rating in the overall sample.

The potential impact of demographic and clinical correlates on the ability to accurately self‐report MDC was also analyzed. First, for *appreciation* and *reasoning*, increased age was associated with poorer ability to accurately self‐report true MDC performance. This could be due to older adults’ greater desire for independence, decreased likelihood to admit a problem, or even anosognosia (Fox, Mitchell, & Booth‐Jones, 2006; Meyers et al., 2004). Older adults are also likely to have previously undetected age‐related cognitive decline and lower education, both of which may influence their ability to self‐recognize MDC (Han et al., 2016). Second, prior chemotherapy use demonstrated a higher odds of disagreement between the self‐report and CCTI measures for Standard 3. This is not unexpected due to common cognitive side effects of chemotherapy (Du, Xia, & Hardy, 2010; Hurria et al., 2006; Vannorsdall, 2017; Vega, Dumas, & Newhouse, 2017; Wefel, Saleeba, Buzdar, & Meyers, 2010; Wefel & Schagen, 2012). Finally, the predictors must be viewed in the context of our high rate of intact self‐ratings in the sample. This may indicate that age and past chemotherapy are actually even stronger predictors of MDC than indicated in this sample.

### 4.1 Clinical implications

Results from this study have several clinical implications for oncology practice. Persons with metastatic cancer are faced with important decisions regarding treatment, end of life, and palliative care. These decisions have potential to impact not only their own physical and emotional well‐being, but also the well‐being of their families and/or caregivers. These results indicate that simply asking a person with metastatic cancer whether they have confidence in their MDC is not enough to be assured of their true MDC abilities. In addition, even those patients who present clinically with seemingly intact MDC may actually be impaired when an objective assessment is used. A more detailed assessment of capacity by the treating physician or consulting psychiatrist or psychologist is needed. If warranted, formal assessment via validated measures of capacity and/or neuropsychological testing, in our opinion, could greatly improve treatment decision making and should be considered despite the slight increased time required. While patient autonomy is a clinical priority, protection of the self‐interest of impaired patients is equally important. Lack of proper assessment of MDC can result in violation of these interests. Furthermore, it is important to note that patients and clinicians may be evaluating MDC differently. Different individuals may place more value on memory‐related versus executive tasks to evaluate MDC (Earnst, Marson, & Harrell, 2000), which could explain disagreement between patient and clinician. Standards for clinical practice should reflect the complexity of both the individual MDC process and the process of ascertaining MDC in the clinic.

Given that different MDC standards vary in level of cognitive complexity, clinical evaluation should take this variability into consideration, especially as treatment options and decisions become riskier and more complex. In older adults and/or those undergoing chemotherapy, neuropsychological workup may be warranted. However, the depth of neuropsychological evaluation may be fluid and based as a function of decision risk. Research into the balance between ensuring proper MDC and the promptness which some cancer care decisions require is warranted. Additionally, development of MDC instruments that can be quickly and easily administered is needed. These instruments could help to ensure that the ethical and legal rights of patients are protected without burdening clinicians.

### 4.2 Study strengths and limitations

This study has several strengths. First, this study is the first of its kind to examine inter‐rater reliability between a self‐report and objective measure of MDC. Additionally, the sample size of 157 is relatively large given the patients in this sample are terminally ill. Finally, this dataset contains extensive relevant cognitive and clinical data for evaluation of predictors of agreement between the two measures.

Despite its strengths, this study is not without limitations. First, we cannot determine specifics related to cancer treatment, such as type of chemotherapy, dose, or duration, that may affect MDC. Second, although the overall sample size was relatively large for a study assessing metastatic cancer patients, the number of individuals in several subgroups was sufficiently small to preclude subgroup analyses. Moreover, all participants in this study scored above a 3 on the Six‐Item Screener, corresponding to little or no cognitive impairment. However, cutoff scores on cognitive testing should not be independently used for determining MDC, and the presence of an organic cognitive disorder, such as dementia, may not necessarily indicate impaired MDC, though it does render the assessment of MDC more difficult (Appelbaum, 2010; Walaszek, 2009). In the current study, we aimed to assess the accuracy of self‐report versus objective measures of MDC in less cognitively impaired individuals where assessment is less difficult, but assessment of agreement among individuals with cognitive impairment is important and future studies should examine the impact of cognitive impairment on agreement between self‐report and objective measures of MDC. Additionally, sample size limited the assessment of many neuropsychological predictors. We assessed a memory task (HVLT total recall t‐score), an executive functioning task (TRAILS B t‐score), and a variable indicating the total number of impaired neuropsychological tests for each participant. Future larger studies should examine several neuropsychological tests and domains as predictors. We did not examine agreement longitudinally, which may change over time. Moreover, the CCTI objectively assesses MDC but is not a universally used measure and is not meant to be a substitute for clinical judgment at this time.

## 5 CONCLUSION

Self‐reported MDC often disagrees with standardized assessments of capacity among metastatic cancer patients, regardless of site. This emphasizes the need for detailed capacity determination by the treating clinician or consultant, supplemented by objective measures of MDC if necessary. Particular attention is warranted with older age and prior receipt of chemotherapy, which decrease the likelihood for self‐recognition of lack of MDC. Further research is needed to elucidate longitudinal trends in subjective assessment of MDC, other potential predictors of agreement, and to determine additional factors influencing age and chemotherapy's effects on agreement.

## AUTHOR'S CONTRIBUTIONS

Mackenzie E. Fowler contributed to the analysis and interpretation of data, statistical analysis, and drafting/revising the manuscript for content. Dario A. Marotta contributed to the drafting/revising the manuscript for content. Richard E. Kennedy contributed to the analysis and interpretation of data and drafting/revising the manuscript for content. Adam Gerstenecker contributed to the interpretation of data and drafting/revising the manuscript for content. Meredith Gammon contributed to the obtaining of data and drafting/revising the manuscript for content. Kristen Triebel contributed to the obtaining of data, interpretation of data, and drafting/revising the manuscript for content. The corresponding author affirms that all persons listed as authors contributed significantly to the production of this manuscript. All contributors are legitimate authors.

## FUNDING

This study was funded by the American Cancer Society (MRSG‐14‐204‐01) Triebel, PI. Dr. Gerstenecker's effort on this project was supported by a K23 Mentored Patient‐Oriented Research Career Development Award (K23HD091849).

## CONFLICTS OF INTEREST

The authors report no conflicts of interest.

### TRANSPARENT PEER REVIEW

The peer review history for this article is available at https://publons.com/publon/10.1002/brb3.2303

## Supporting information

Supporting Information
