# Supplementary material for: Reliability of self‐report versus the capacity to consent to treatment instrument to make medical decisions in brain metastasis and other metastatic cancers
Source: Brain Behav. 2021 Oct 2;11(11):e2303. doi: 10.1002/brb3.2303 (PMC8613414; doi:10.1002/brb3.2303)
Supplement: Supplementary file 1 — Supporting Information [file BRB3-11-e2303-s001.docx]

| **Supplementary Table 1. Neuropsychological Characteristics by Objective Medical Decision-Making Capacity Performance and Diagnosis^†^** | | | | | | | | | |
| --- | --- | --- | --- | --- | --- | --- | --- | --- | --- |
| **Variable** | **Overall (n=155)** | | | **Brain Metastasis (n=114)** | | | **Other Metastasis (n=41)** | | |
|  | **Intact** | **Impaired** | **p-value** | **Intact** | **Impaired** | **p-value** | **Intact** | **Impaired** | **p-value** |
| ***Subjective Memory/Cognitive Changes*** | 13 (31.0) | 32 (49.2) | 0.0731 | 5 (22.7) | 18 (40.9) | 0.1778 | 8 (40.0) | 14 (66.7) | 0.0870 |
| ***Subjective Concern with Thinking Abilities*** | 7 (17.1) | 19 (29.7) | 0.1697 | 3 (13.6) | 16 (36.4) | 0.0830 | 4 (21.1) | 3 (15.0) | 0.6948 |
| **Neuropsychological Variables** |  |  |  |  |  |  |  |  |  |
| ***Digit Span Raw*** | 16.2 ± 3.4 | 14.1 ± 3.4 | **0.0003** | 16.0 ± 3.2 | 13.7 ± 3.3 | **0.0007** | 16.6 ± 3.8 | 15.1 ± 3.9 | 0.2304 |
| ***Digit Span Raw Forward*** | 10.0 ± 2.0 | 9.2 ± 2.3 | **0.0312** | 10.1 ± 2.0 | 9.1 ± 2.3 | **0.0254** | 9.9 ± 1.9 | 9.6 ± 2.4 | 0.6851 |
| ***Digit Span Raw Backward*** | 6.1 ± 2.0 | 5.0 ± 2.1 | **0.0014** | 5.8 ± 1.6 | 4.6 ± 1.6 | **0.0004** | 6.8 ± 2.5 | 6.1 ± 3.0 | 0.4556 |
| ***Longest Digit Span Forward*** | 6.7 ± 1.2 | 6.2 ± 1.3 | 0.0628 | 6.8 ± 1.1 | 6.2 ± 1.3 | 0.0554 | 6.6 ± 1.3 | 6.3 ± 1.5 | 0.5377 |
| ***Longest Digit Span Backward*** | 4.7 ± 1.2 | 4.0 ± 0.9 | **0.0011** | 4.5 ± 0.9 | 3.8 ± 0.9 | **0.0058** | 5.0 ± 1.5 | 4.2 ± 0.9 | 0.0764 |
| ***Animals Raw*** | 20.0 ± 5.5 | 15.6 ± 4.3 | **<0.0001** | 19.2 ± 5.6 | 14.8 ± 4.2 | **<0.0001** | 21.6 ± 5.2 | 18.1 ± 3.9 | **0.0200** |
| ***CFL Raw*** | 36.2 ± 13.4 | 26.0 ± 10.2 | **<0.0001** | 35.9 ± 14.4 | 24.3 ± 10.0 | **<0.0001** | 36.7 ± 11.4 | 31.2 ± 9.5 | 0.1026 |
| ***HVLT Total Recall*** | 23.9 ± 5.3 | 19.8 ± 5.7 | **<0.0001** | 23.2 ± 5.3 | 18.9 ± 5.7 | **0.0002** | 25.4 ± 5.1 | 22.5 ± 4.9 | 0.0695 |
| ***HVLT Delayed Raw*** | 8.3 ± 2.8 | 6.6 ± 3.0 | **0.0006** | 8.0 ± 2.8 | 6.2 ± 3.0 | **0.0018** | 9.0 ± 2.8 | 8.1 ± 2.7 | 0.2927 |
| ***HVLT Retention*** | 86.7 ± 21.2 | 80.4 ± 30.2 | 0.1345 | 85.6 ± 21.4 | 76.7 ± 31.0 | 0.0805 | 88.9 ± 21.2 | 92.3 ± 24.6 | 0.6401 |
| ***Trails A Raw*** | 34.6 ± 16.5 | 44.4 ± 26.5 | **0.0074** | 35.6 ± 16.7 | 47.8 ± 29.1 | **0.0078** | 32.8 ± 16.3 | 33.8 ­± 11.1 | 0.8166 |
| ***Trails A Errors*** | 0.2 ± 0.2 | 0.2 ± 0.5 | 0.8196 | 0.2 ± 0.5 | 0.2 ± 0.6 | 0.8259 | 0.2 ± 0.4 | 0.1 ± 0.4 | 0.9500 |
| ***Trails B Raw*** | 114.2 ± 70.3 | 147.3 ± 83.6 | **0.0149** | 118.9 ± 78.0 | 158.2 ± 86.8 | **0.0248** | 105.3 ± 53.6 | 115.6 ± 65.4 | 0.5831 |
| ***Trails B Errors*** | 0.8 ± 1.2 | 0.9 ± 1.4 | 0.7814 | 1.0 ± 1.5 | 1.0 ± 1.5 | 0.9823 | 0.6 ± 0.5 | 0.7 ± 0.9 | 0.6149 |
| ***Digit Symbol Raw*** | 55.9 ± 18.0 | 43.5 ± 16.2 | **<0.0001** | 56.7 ± 17.0 | 42.2 ± 15.9 | **<0.0001** | 54.5 ± 20.0 | 47.3 ± 16.9 | 0.2256 |
| ***WRAT Raw*** | 45.9 ± 7.6 | 44.8 ± 6.5 | 0.3529 | 45.8 ± 7.0 | 45.0 ± 6.9 | 0.6097 | 46.1 ± 9.0 | 43.9 ± 4.9 | 0.3438 |
| ***Subjective FAQ Total*** | 6.1 ± 8.2 | 9.7 ± 10.8 | 0.0715 | 6.1 ± 8.3 | 10.9 ± 10.5 | 0.0747 | 6.1 ± 8.3 | 7.2 ± 11.2 | 0.7273 |
| ***Study Partner FAQ Total*** | 9.1 ± 11.8 | 7.6 ± 9.8 | 0.6344 | 14.3 ± 13.5 | 8.2 ± 9.6 | 0.1452 | 3.8 ± 7.1 | 5.1 ± 11.0 | 0.7669 |
| ***HADS Anxiety Total*** | 6.3 ± 3.9 | 6.5 ± 5.1 | 0.8070 | 7.1 ± 4.0 | 7.0 ± 4.6 | 0.9335 | 5.5 ± 3.8 | 5.6 ± 6.1 | 0.9699 |
| ***HADS Depression Total*** | 4.7 ± 3.0 | 5.5 ± 4.2 | 0.2601 | 4.6 ± 2.8 | 6.3 ± 4.5 | 0.0792 | 4.8 ± 3.4 | 3.8 ± 2.9 | 0.3595 |
| **Total Neuropsychological Tests Impaired** | 1.3 ± 1.7 | 2.7 ± 2.4 | **0.0002** | 1.6 ± 1.8 | 3.1 ± 2.5 | **0.0026** | 0.9 ± 1.5 | 1.5 ± 1.5 | 0.1893 |
| ^†^Intact vs. Impaired status determined via performance on standards 3-5 on the Capacity to Consent to Treatment Index (CCTI) | | | | | | | | | |

**Supplementary Figure 1. Current Medical Decision Making Capacity Rating Self-Report Version (CMDC).**

**
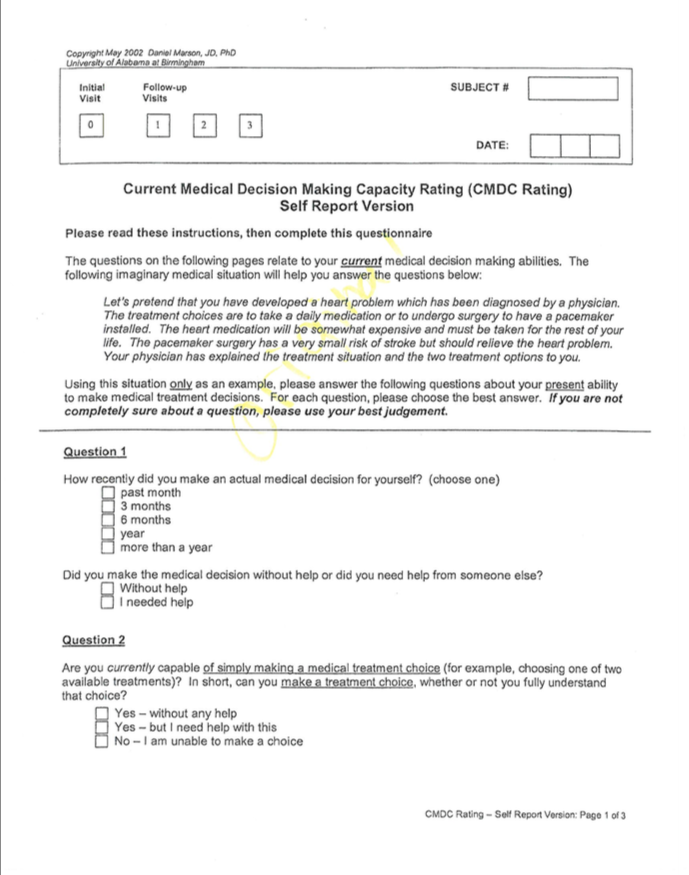
**

**
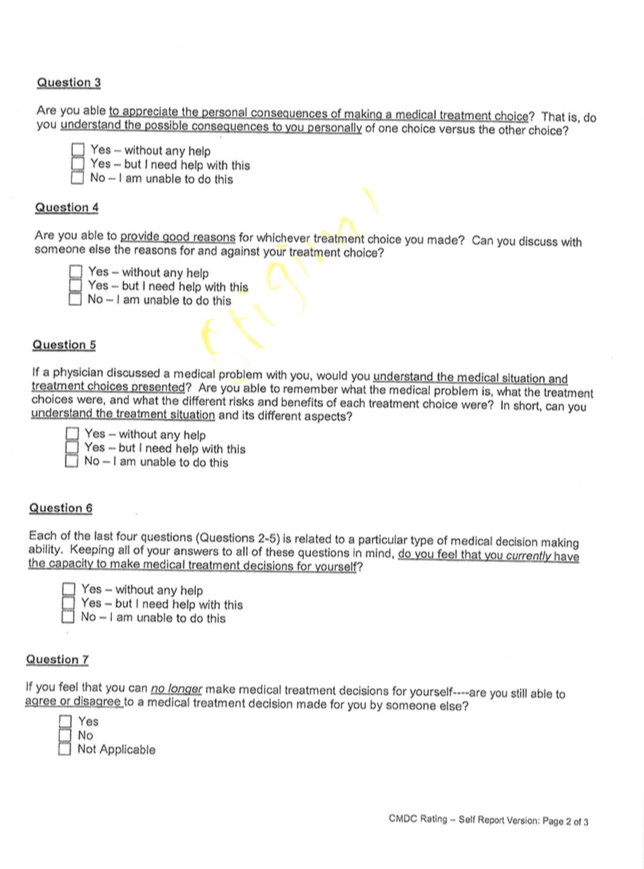
**

**
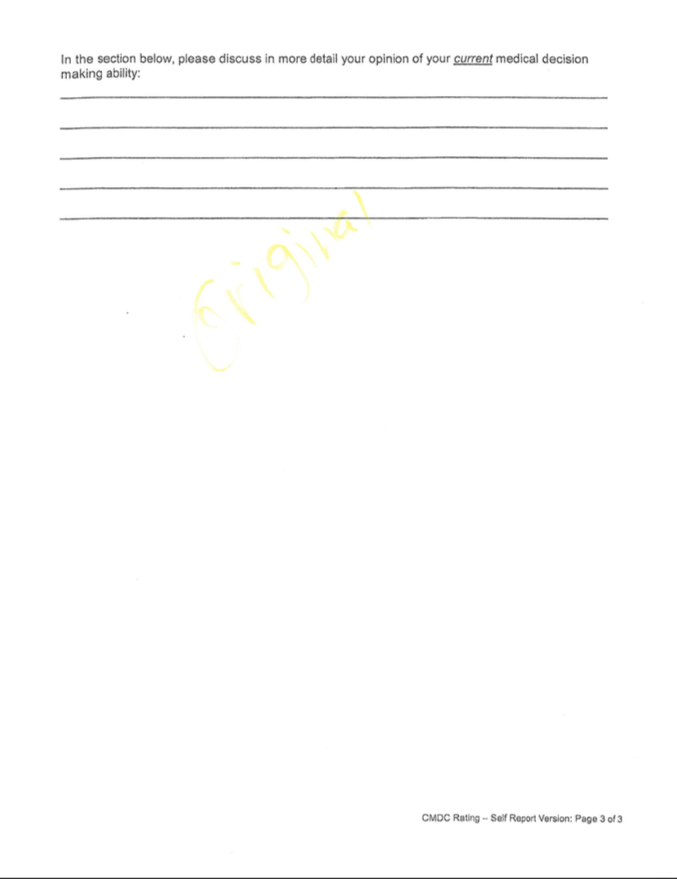
**
